# Effect of Trunk Rotation and Sex Differences on Lung Volume and Respiratory Muscle Strength in Healthy Young People

**DOI:** 10.7759/cureus.105165

**Published:** 2026-03-13

**Authors:** Miki Takahata, Miho Osawa, Mizuki Hoshina, Michiyasu Yamaki, Toshiaki Sato

**Affiliations:** 1 Department of Occupational Therapy, Faculty of Health Science, Yamagata Prefectural University of Health Sciences, Yamagata, JPN; 2 Department of Rehabilitation, Akita Cerebrospinal and Cardiovascular Center, Akita, JPN; 3 Department of Rehabilitation, Okitama Public General Hospital, Yamagata, JPN; 4 Department of Cardiology, Yamagata National Hospital, Yamagata, JPN

**Keywords:** lung volume, respiratory muscle strength, sex differences, spirometry, trunk rotational posture

## Abstract

Background

Posture and trunk movements influence respiratory mechanics by altering rib cage mobility and thoracoabdominal motion. Trunk rotation frequently occurs during activities of daily living and may affect lung volumes and respiratory muscle function. Anatomical and physiological differences between males and females may also influence respiratory responses to posture. However, the combined effects of trunk rotation and biological sex on pulmonary function and respiratory muscle strength remain unclear. This study aimed to evaluate the effects of trunk rotation and sex differences on lung volume parameters and respiratory muscle strength in healthy young adults.

Methodology

A total of 20 healthy young adults (9 males and 11 females; mean age = 22 ± 1 years) participated in this study. Participants performed pulmonary function tests in the sitting position at the resting posture or in the sitting position with 30° trunk rotation. Lung function parameters, including vital capacity (VC), inspiratory capacity (IC), tidal volume (VT), expiratory reserve volume (ERV), inspiratory reserve volume (IRV), forced vital capacity (FVC), and forced expiratory volume in one second (FEV1.0), were measured using spirometry. Respiratory muscle strength was assessed using maximal inspiratory pressure (PImax) and maximal expiratory pressure (PEmax). Two-way repeated-measures analysis of variance was used to evaluate the effects of posture and sex.

Results

Significant main effects of sex were observed for several respiratory variables, including FVC, FEV1.0, VC, ERV, PImax, and PEmax, with males showing higher values than females. Significant main effects of posture were also observed for FVC, FEV1.0, VC, ERV, PImax, and PEmax, with lower values in the rotational posture than in the resting posture. A significant interaction between posture and sex was found for PEmax, with a greater decrease in males during trunk rotation. In contrast, PImax decreased significantly in the rotational posture only in females.

Conclusions

Trunk rotation in the sitting position reduces several lung volume parameters and respiratory muscle strength in healthy young adults, and the effects may differ between males and females. In particular, trunk rotation may affect forced expiration more in males and inspiratory muscle strength more in females. These findings provide fundamental data on the interaction between posture and sex in respiratory mechanics and may provide useful insights for respiratory assessment and rehabilitation strategies involving trunk movement.

## Introduction

The structure and function of the respiratory system differ between males and females. Females are characterized by a smaller-sized rib cage and narrower airways relative to lung size compared to males [1-4]. Additionally, there are sex differences in rib cage geometry, with females demonstrating a greater inclination of the ribs than males [5,6]. These anatomical differences influence thoracic movement and respiratory muscle mechanics. Consequently, it has been reported that females have a higher thoracic contribution to thoracoabdominal motion during respiration compared to males [7-10]. These anatomical and physiological differences also affect exercise performance, with females tending to experience increased respiratory rates and symptoms such as shortness of breath or dyspnea during exercise due to higher respiratory resistance.

Posture and trunk movements (including rotation) are known to affect lung function by altering the movement of the rib cage and abdomen during ventilation. In particular, trunk rotation plays a crucial role in both functional movement and respiratory mechanics. Trunk rotation primarily occurs around the spine, affects rib cage mobility, and influences lung volume. Improved trunk mobility promotes chest wall expansion, increases lung volume, and improves ventilation efficiency [11]. In contrast, restriction of trunk rotation limits thoracic movement and has a negative impact on respiratory function. This relationship is particularly relevant in clinical populations such as patients with scoliosis, chronic obstructive pulmonary disease, and neuromuscular disorders, where postural asymmetry or musculoskeletal dysfunction impacts breathing mechanics. Understanding the relationship between trunk rotation and lung volume is essential for effective physical therapy and rehabilitation strategies, ultimately optimizing exercise performance. Changes in posture, such as increasing trunk inclination from sitting to supine, have been shown to reduce rib cage displacement and increase abdominal muscle activity, thereby altering minute ventilation. Anatomical sex-related differences suggest that the effects of posture on respiratory mechanics may also differ between males and females. Mendes et al. reported sex differences in the effects of posture on respiratory function [12]. However, to date, there are limited reports addressing the combined influence of biological sex and posture on pulmonary function.

Furthermore, trunk rotational posture is a frequently occurring position in daily life. Previous studies have shown that trunk rotation can significantly reduce lateral thoracic movement at the axillary level [13]. Given that females generally exhibit a higher thoracic contribution during respiration, the effects of rotational posture on respiratory function may be greater in females than in males.

This study aimed to evaluate the effects of a 30° trunk rotation in the sitting position and biological sex on pulmonary function and respiratory muscle strength in healthy young adults. By comparing lung volume and respiratory pressure parameters between males and females under resting and rotated postures, the study seeks to clarify posture-related and sex-specific differences in respiratory function relevant to daily living and rehabilitation. The clarification of postural effects and sex differences on respiratory function will provide insight into the symptoms of breathlessness during activities of daily living. The clarification of these posture-related changes in respiratory indices and the effects of sex differences is crucial for providing movement guidance and rehabilitation tailored to the subject. This study of healthy young people provides fundamental data on the interaction of posture and sex differences during resting respiration.

This article was previously posted to the Research Square preprint server in August, 2021.

## Materials and methods

Participants

A total of 20 healthy young people (9 males and 11 females; mean age = 22 ± 1 years for both sexes) were enrolled in this study. The inclusion criteria were nonsmokers with no cardiac or pulmonary diseases. All participants were nonathlete students in the university’s health sciences department. They had no evidence of alterations of the physiological curvature of the spine. We conducted the priori power analysis for repeated-measures analyses of variance (ANOVA) by G*power 3.1 with the expected effect size of 0.4 and a significance level of 0.05 at the desired power of 0.8.

Procedures

Participants’ body composition was assessed by the bioelectrical impedance method using InBody 270 (Inbody 270; Inbody, Tokyo, Japan).

Participants performed pulmonary function tests in the sitting position at rest (the resting posture) or in the sitting position with 30° trunk rotation (the rotational posture). The range of motion of trunk rotation was measured based on the angle formed by the intersection of the line connecting the posterior superior iliac spines on both sides and the line connecting the acromion of the scapula [14]. The trunk was rotated 30° to the right side. Participants were instructed to maintain each posture until the trunk rotation angle was confirmed and breathing was stable. After posture stabilization, pulmonary function measurements were performed. The order of posture testing (resting posture or rotational posture) was randomized for each participant. Rest periods were provided between measurements to minimize fatigue. Pulmonary function was assessed using a calibrated spirometer (HI-801; CHEST M.I., Tokyo, Japan), according to an American Thoracic Society/European Respiratory Society (ATS/ERS) statement about pulmonary function tests [15]. Variables such as vital capacity (VC), inspiratory capacity (IC), tidal volume (VT), expiratory reserve volume (ERV), inspiratory reserve volume (IRV), forced vital capacity (FVC), forced expiratory volume in one second (FEV1.0), and percentage of forced expiratory volume in one second (FEV1.0%) were measured. To determine respiratory muscle strength, the maximal inspiratory pressure (PImax) and maximal expiratory pressure (PEmax) were measured using a mouth pressure meter (IOP-01; Kobata, Osaka, Japan), in accordance with an ATS/ERS statement about respiratory muscle testing [16]. The maximum values of the measurements for each of the three maneuvers, varying by less than 20%, were recorded. Each measurement was performed with a rest period considering fatigue.

Statistical analysis

Respiratory variables were calculated as means ± standard deviations. We used SPSS Statistics software, version 24.0 (IBM Corp., Armonk, NY, USA), to analyze the data. We performed two-way ANOVA with repeated measures to compare the effects across posture and sex differences on each variable. Bonferroni’s correction was used for the post hoc analysis. The level of significance was set at a p-value <0.05 for all statistical comparisons. Effect sizes were interpreted according to Cohen’s criteria (small ≥0.01, medium ≥0.06, large ≥0.14).

Ethical approval and consent to participate

This study’s ethical approval was granted by the Ethics Review Board of Yamagata Prefectural University of Health Sciences, Yamagata, Japan (#1801-23), and this study was performed in accordance with the recommendations of the Ethics Review Board of Yamagata Prefectural University of Health Sciences. All participants provided written informed consent to participate, in accordance with the Declaration of Helsinki.

## Results

The characteristics of the 20 participants are shown in Table 1. Participants showed significant sex differences in parameters except for age.

**Table 1:** Physiological characteristics of participants. Data are presented as mean ± standard deviations. **: p < 0.01, ***: p < 0.001. BMI: body mass index; SMI: skeletal muscle mass index

	All (n = 20)	Male (n = 9)	Female (n = 11)	t	d
Age (year)	22 ±1	22 ± 1	22 ± 1	-0.68	0.33
Height (cm)	163.5 ± 8.4	170.8 ± 5.8	157.5 ± 4.6***	5.30	2.57
Weight (kg)	57.4 ± 12.9	68.6 ± 11.1	48.3 ± 3.6***	5.36	2.58
BMI (kg/m^2^)	21.3 ± 3.4	23.5 ± 3.6	19.4 ± 1.5**	3.34	1.55
SMI (kg/m^2^)	6.7 ± 1.3	7.9 ± 0.8	5.6 ± 0.4***	8.16	3.76

The participants performed the pulmonary function test in the rest or 30° trunk rotation in the sitting position. On testing, the values of VC, IC, ERV, IRV, FVC, and FEV1.0 were significantly higher for males than for females in both postures (Table 2).

**Table 2:** Differences in respiratory and respiratory muscle variables during the rest or rotational posture in the sitting position between male and female participants. All data are presented as mean ± standard deviations. VC: vital capacity; IC: inspiratory capacity; VT: tidal volume; ERV: expiratory reserve volume; IRV: inspiratory reserve volume; FVC: force vital capacity; FEV1.0: forced expiratory volume in one second; FEV1.0%: forced expiratory volume % in one second; PImax: maximal inspiratory pressure; PEmax: maximal expiratory pressure; n.s.: not significant

		Male	Female	t	P-value	d
VC (L)	Rest	4.7 ± 0.6	3.0 ± 0.4	6.97	<0.001	3.13
	Rotation	4.3 ± 0.7	2.7 ± 0.5	6.06	<0.001	2.72
IC (L)	Rest	2.6 ± 0.4	1.7 ± 0.4	5.33	<0.001	2.39
	Rotation	2.6 ± 0.4	1.7 ± 0.4	4.49	<0.001	2.02
TV (L)	Rest	0.8 ± 0.3	0.7 ± 0.4	0.64	n.s.	0.01
	Rotation	0.8 ± 0.3	0.7 ± 0.3	0.56	n.s.	0.06
ERV (L)	Rest	2.0 ± 0.4	1.3 ± 0.4	4.22	<0.001	1.90
	Rotation	1.7 ± 0.3	1.0 ± 0.3	4.79	<0.001	2.15
IRV (L)	Rest	1.8 ± 0.4	1.0 ± 0.3	5.52	<0.001	2.48
	Rotation	1.8 ± 0.4	1.0 ± 0.3	5.79	<0.001	2.60
FVC (L)	Rest	4.7 ± 0.6	3.1 ± 0.4	7.06	<0.001	3.18
	Rotation	4.4 ± 0.7	2.7 ± 0.5	6.37	<0.001	2.86
FEV1.0 (L)	Rest	4.1 ± 0.5	2.8 ± 0.4	6.55	<0.001	2.93
	Rotation	3.8 ± 0.6	2.5 ± 0.5	5.89	<0.001	2.63
FEV1.0% (%)	Rest	87.2 ± 4.6	90.4 ± 4.7	-1.50	n.s.	0.94
	Rotation	87.1 ± 4.4	89.9 ± 4.6	-1.40	n.s.	0.56
PImax (cmH_2_O)	Rest	89.5 ± 32.1	65.6 ± 21.5	1.99	n.s.	0.89
	Rotation	86.1 ± 28.9	59.4 ± 16.4	2.61	0.02	1.17
PEmax (cmH_2_O)	Rest	101.5 ± 25.9	62.7 ± 14.8	4.20	0.002	1.89
	Rotation	89.1 ± 29.0	58.5 ± 13.8	3.12	0.006	1.40

**Figure 1:**
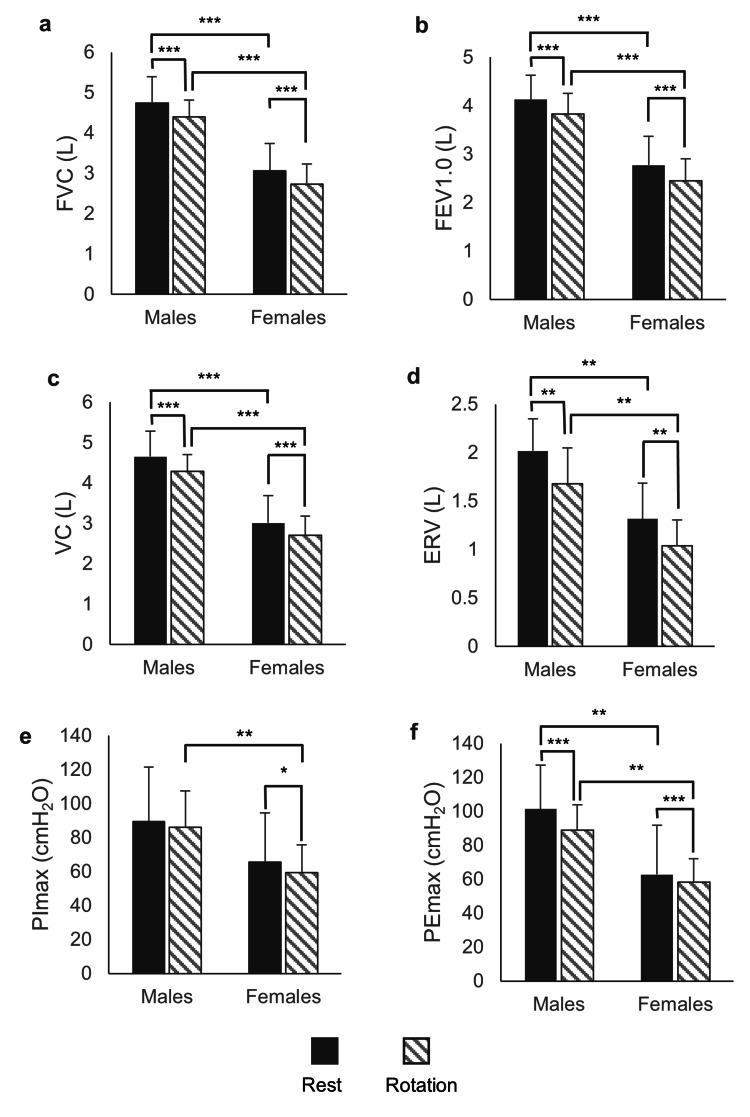
Differences in respiratory variables during the resting or 30° rotation of the thoracic in the sitting position. (a) forced vital capacity (FVC), (b) forced expiratory volume in one second (FEV1.0), (c) vital capacity (VC), (d) expiratory reserve volume (ERV), (e) maximal inspiratory pressure (PImax), and (f) maximal expiratory pressure (PEmax). *: p < 0.05; **: p < 0.01; ***: p < 0.001.

Figure 1 shows the results of the two-way ANOVA with repeated measures and the post hoc analysis. There were significant main effects of sex differences on FVC (F1, 18 = 45.8, p < 0.001, ηn² = 0.72; large), FEV1.0 (F1, 18 = 45.8, p < 0.001, ηn² = 0.69; large), VC (F1, 18 = 44.6, p < 0.001, ηn² = 0.71; large), ERV (F1, 18 = 25.0, p < 0.001, ηn² = 0.58; large), PImax (F1, 18 = 5.3, p = 0.034, ηn² = 0.29; large), and PEmax (F1, 18 = 13.6, p = 0.002, ηn² = 0.43; large). These variables had higher values for males than for females in both the resting and rotational postures.

There were significant main effects of posture on FVC (F1, 18 = 45.8, p < 0.001, ηn² = 0.82; large), FEV1.0 (F1, 18 = 40.62, p < 0.001, ηn² = 0.69; large), VC (F1, 18 = 44.6, p < 0.001, ηn² = 0.73; large), ERV (F1, 18 = 25.0, p < 0.001, ηn² = 0.53; large), PImax (F1, 18 = 7.3, p = 0.002, ηn² = 0.23; large), and PEmax (F1, 18 = 32.1, p < 0.001, ηn² = 0.64; large). For males and females, these values were significantly lower in the rotational posture than in the resting posture. Moreover, there was a significant interaction between sex and posture in PEmax (F1, 18 = 7.5, p = 0.0013, ηn² = 0.30; large): the PEmax value was decreased more in males than in females by the rotational posture. In contrast, the PImax value decreased significantly in the rotational posture only in females (p = 0.024, d = 0.33).

## Discussion

In the present study, we examined the effects of trunk rotation in the sitting position and sex differences for healthy young people by comparing respiratory variables. This study indicated that the effects of rotational posture on PImax and PEmax may be different between males and females. These findings may provide important insights into sex differences in respiration in daily living.

VC and FVC exhibited significant decreases in the rotational posture in comparison with the resting posture in the sitting position. Lee et al. reported reduced motion of the rib cage at the axilla during respiration with spinal rotation [13]. The rotational posture causes a range of motion decrease in the rib cage, changing its articulations and intercostal muscle activities, and requires increased abdominal motion. These thoracoabdominal motion changes may have induced the decreases in VC and FVC.

Additionally, ERV and FEV1.0 also decreased during respiration in the rotational posture in comparison with the resting posture. Moreover, in the rotational posture, males may have a greater reduction in PEmax than females. It was previously reported that obesity and external pressure on the rib cage reduce ERV [17]. In this study, the trunk rotation posture significantly reduced the forced expiratory muscle strength. The agonist muscles for trunk rotation include the external and internal oblique abdominal muscles, which are the most active during forced expiration. Therefore, the results of that study suggest that rotational posture may limit the activity of the muscles used in forced expiration. Chest wall kinematics during respiration are significantly influenced by sex differences [9,10]. There are differences in the relative contribution of the rib cage and abdomen during respiration. Bellemare et al. reported that the differences in the composition of thoracic dimensions and configuration between males and females produce differences in the contribution of the rib cage and abdomen during ventilation [7]. The significant interaction between posture and sex for PEmax may suggest that males may be more affected by limitations of trunk rotation during forced expiration due to the higher contribution of the abdomen during respiration.

PImax significantly decreased in the rotational posture in comparison with the rest posture in females, whereas it did not change in males. Females have a higher thoracic contribution to thoracoabdominal motion during respiration compared to males [7]. The rotational posture restricts the motion of the rib cage during respiration. Therefore, the restriction of rib cage expansion during inspiration may have reduced inspiratory muscle strength in females. We deduced that rotational posture, which restricted the range of motion of the rib cage, affected inspiratory muscle strength in females.

These findings may have practical implications for rehabilitation and daily activities involving trunk rotation, such as reaching, dressing, and turning while sitting. Understanding how trunk rotation affects respiratory mechanics may help clinicians consider postural alignment during respiratory assessment and rehabilitation. In addition, sex-related differences in respiratory responses to trunk rotation should be considered when designing individualized rehabilitation programs.

Several limitations should be acknowledged. First, the sample size was relatively small, which may limit the generalizability of the findings. Second, the participants were healthy young adults, and therefore, the results may not be applicable to elderly individuals or clinical populations. Third, body characteristics such as body mass index, body weight, and abdominal circumference, which may influence pulmonary function, were not controlled in the present study. Most participants also had relatively slender body types, which may further limit generalizability. Future studies with larger and more diverse populations are needed to confirm these findings.

## Conclusions

This study showed that trunk rotation decreases lung capacity and respiratory muscle strength and that there may be sex differences in the effect of trunk rotation posture on respiratory muscle strength. Particularly, this study suggests that the rotational posture affects forced exhalation in males and inspiration in females. These findings from this study made several contributions to provide fundamental data on the interaction of posture and sex differences during resting respiration. The clarification of these posture-related changes in respiratory indices and the effects of sex differences is crucial for providing movement guidance and rehabilitation tailored to the subject. Further studies of elderly patients and those with respiratory diseases are needed to clarify the effects of posture and sex on respiration.
